# The Regulatory Role of Ferroptosis in Bone Homeostasis

**DOI:** 10.1155/2022/3568597

**Published:** 2022-07-13

**Authors:** Yuan Xiong, Lang Chen, Ze Lin, Yiqiang Hu, Adriana C. Panayi, Wu Zhou, Yun Sun, Faqi Cao, Guodong Liu, Guangdong Dai, Bobin Mi, Guohui Liu

**Affiliations:** ^1^Department of Orthopaedics, Union Hospital, Tongji Medical College, Huazhong University of Science and Technology, Wuhan 430022, China; ^2^Hubei Province Key Laboratory of Oral and Maxillofacial Development and Regeneration, Wuhan 430022, China; ^3^Department of Plastic Surgery, Brigham and Women's Hospital, Harvard Medical School, Boston, MA 02152, USA; ^4^Department of Neurosurgery, Union Hospital, Tongji Medical College, Huazhong University of Science and Technology, Wuhan 430022, China; ^5^Medical Center of Trauma and War Injuries, Daping Hospital, Army Medical University, Chongqing 400042, China; ^6^Pingshan District People's Hospital of Shenzhen, Pingshan General Hospital of Southern Medical University, Shenzhen, Guangdong, China 518118

## Abstract

Ferroptosis is an iron-dependent form of programmed cell death and an important type of biological catabolism. Through the action of divalent iron or ester oxygenase, ferroptosis can induce lipid peroxidation and cell death, regulating a variety of physiological processes. The role of ferroptosis in the modulation of bone homeostasis is a significant topic of interest. Herein, we review and discuss recent studies exploring the mechanisms and functions of ferroptosis in different bone-related cells, including mesenchymal stem cells, osteoblasts, osteoclasts, and osteocytes. The association between ferroptosis and disorders of bone homeostasis is also explored in this review. Overall, we aim to provide a detailed overview of ferroptosis, summarizing recent understanding on its role in regulation of bone physiology and bone disease pathogenesis.

## 1. Introduction

Ferroptosis is a form of cell death that was only recently defined by [1]. who proposed the concept in 2012 to describe a nonapoptotic type of cell death which is iron-dependent and is characterized by an accumulation of reactive oxygen species (ROS). Ferroptosis is closely related to a variety of metabolic disorders, tumors, and injuries [2–4]. During ferroptosis, the most susceptible lipids to peroxidation are polyunsaturated fatty acids (PUFAs).

In cell physiology, an increase of polyunsaturated fatty acids (PUFAs) on the cell membrane enhances the fluidity of the cell membrane, which indirectly increases the migratory ability of the cell [5]. Therefore, the increase in PUFAs is an important hallmark in the process of cell evolution. However, introduction of PUFAs also endangers cell survival. Hydrogen molecules produced during the dissociation of PUFAs can react with oxide and ferrous ions in the surrounding environment, resulting in the accumulation of peroxide and subsequent cell damage [6]. Normally, cells use PUFAs efficiently without causing cell damage by employing glutathione peroxidase 4 (GPX4) signaling which partially decreases the levels of PUFAs [7]. Mechanistically, GPX4 uses its catalytic activity to weaken the toxicity of lipid peroxides and maintain the homeostasis of lipid bilayer. Recent evidence identified the regulatory role of GPX4 and ferroptosis in multiple pathological processes. Currently, ferroptosis has attracted accumulative focus in studies on a wide range of diseases. Plenty of evidences have demonstrated that the pathological process of ferroptosis involves the excessive production of ROS, followed by abnormal activation of lipid peroxidation in an iron-dependent manner, accompanied with a marked elevated uptake of PUFAs into the cellular membrane. The unique characteristics of ferroptosis make it complicated related to several biological processes.

Bone homeostasis is a physiological process regulated by bone related-stem cells, osteoblasts, osteoblasts, and osteocytes [8]. During bone remodeling, osteocytes, osteoblasts, and osteoclasts interact with one another in a paracrine manner and regulate angiogenesis in the bone marrow to maintain bone homeostasis [9]. Research has demonstrated the crucial role of ferroptosis in regulating the survival of bone-related cells and identified oxidative stress as an important factor in cell death [10, 11]. However, the exact mechanism of ferroptosis in bone homeostasis regulation remains largely unknown, and it is yet unclear whether ferroptosis is a driver or a passenger event in bone homeostasis.

Herein, we aim to review the recent literature on the subject to explore the underlying mechanisms of ferroptosis and its roles in different bone-related cells, including mesenchymal stem cells, osteoblasts, osteoclasts, and osteocytes. We summarize the recent findings on the role of ferroptosis in regulation of bone physiology and osteopathogenesis.

## 2. Ferroptosis

As previously mentioned, ferroptosis was first proposed in 2012 but was redefined as a mode of programmed cell death closely related to cell oxidative disturbance by the Nomenclature Committee on Cell Death in 2018 [12]. Compared to other classic forms of cell death, ferroptosis is characterized by the accumulation of iron-dependent lipid ROS. Ferroptosis occurs following a depletion of glutathione (GSH), subsequent decrease in the activity of glutathione peroxidase 4 (GPx4), and inhibition of lipid oxide metabolism as this is a GPX4-dependent reaction. Following this, divalent iron ions oxidize the lipid to produce ROS leading to ferroptosis [13].

Susceptibility to ferroptosis is closely related to multiple biological processes, including iron and PUFA metabolism and biosynthesis of GSH, phospholipid, nicotinamide adenine dinucleotide phosphate hydrogen (NADPH), and coenzyme Q10 [13]. It has also been linked to the pathological cell death seen in mammalian degenerative diseases, such as tumors, stroke, cerebral hemorrhage, traumatic brain injury, and renal failure [14, 15].

### 2.1. The Characteristics of Ferroptosis

During ferroptosis, a large number of iron ions are deposited in the dead cells, lipid peroxidation occurs intracellularly, ROS levels increase significantly, and proteins that regulate iron homeostasis and lipid peroxidation metabolism are altered [16]. Microscopically, the mitochondrial membrane shrinks, the mitochondrial crest decreases or disappears, and the outer membrane is broken, although the morphological changes of the nucleus are not as obvious (Table 1).

### 2.2. Underlying Mechanisms of Ferroptosis

**Figure 1:**
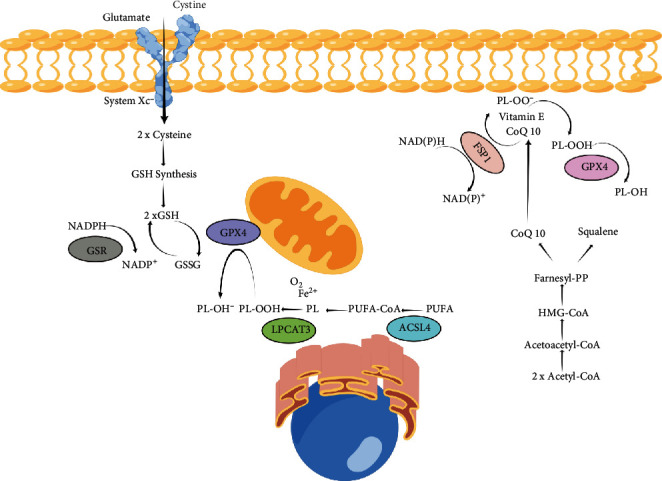
The molecular mechanism of ferroptosis.

Investigations on the regulation of ferroptosis have mainly focused on system Xc^−^ and GSH metabolism, regulation of GPX4 activity, and ROS production (Figure 1). System Xc^−^, which comprises of SLC3A2 and SLC7A11 dimers, has been reported in various cells as a promising target for ferroptosis induction [17–19]. System Xc^−^ is embedded into the cell membrane and as an effective cystine/glutamate antiporter system regulates the transport of cysteine and glutamate [20]. Glutamate is transferred outside the cell, while simultaneously, cystine is imported into the cell where it participates in the generation of GSH and thereby prevents ferroptosis [21]. A recent study reported that IFN-*γ* was capable of suppressing the expression of SLC3A2 and SLC7A11 via activation of JAK/STAT signaling, and repression of system Xc^−^ could induce ferroptosis in hepatocellular carcinoma cells [22]. Similarly, as a tumor suppressor gene, p53 was demonstrated to inhibit cystine uptake by downregulating the expression of SLC7A11, which could decrease the activity of GPX4 and reduce the antioxidant ability of the cells ultimately inducing ferroptosis [23].

Furthermore, GPX4 is considered a crucial molecule in the regulation of ferroptosis [24]. The basis of ferroptosis is the presence of free iron in the cells. The Fenton reaction between iron ions and ROS leads to peroxidation of PUFAs and formation of lipid peroxides, resulting in damage to the cell membrane [25]. GPX4 is able to ameliorate the toxicity of lipid peroxides via its catalytic activity and maintain the homeostasis of the lipid bilayer. Prior studies have shown that RSL3, an inhibitor of GPX4, can covalently bind to GPX4 and inactivate it, ultimately leading to the accumulation of intracellular peroxide and induction of ferroptosis [26].

In addition, the ROS-mediated pathway is a critical mechanism of ferroptosis. As induction of ferroptosis leads to the increase of intracellular lipid ROS, theoretically, lipid antioxidants may be promising antiferroptosis agents [27]. Mitochondria, an organelle with abundant iron and ROS production, are considered to be an important location of the occurrence of ferroptosis.

### 2.3. Mitochondrial Dysfunction Regulates Ferroptosis

Given the important role of mitochondria in ROS generation, their function is critical in ferroptosis [28]. Prior research revealed that complete inhibition of mitochondrial function could significantly decrease cell sensitivity to ferroptosis under cysteine-deprivation conditions [29]. Furthermore, Gaschler et al. [30] reported that partially decreased functioning of mitochondria could restore cell sensitivity to ferroptosis, findings which highlight mitochondria's significant function in initiating ferroptosis. Suppression of the tricarboxylic acid (TCA) cycle and electron transport chain (ETC) was also demonstrated to inhibit ferroptosis, which is consistent with the role of mitochondria in ROS generation [29, 30]. Several enzymes in the TCA cycle are critical for inducing ferroptosis [31]. For instance, a recent study showed that deprivation of fumarate hydratase in renal cancer cells could increase cell tolerance to ferroptosis [32]. Moreover, disruption of the TCA cycle was capable of suppressing lipid peroxidation and ferroptosis [33]. Consistent with the significant role of this process in mediating ferroptosis, suppression of the ETC was found to inhibit ROS accumulation and the induction of ferroptosis in response to either cysteine deprivation or erastin (a ferroptosis inducer) treatment [29].

## 3. Ferroptosis and Bone Homeostasis

Homeostasis is a complicated balance that is crucial for cells to maintain their normal physiological functions [30]. A tight balance between energy input and consumption is important for cell homeostasis. During metabolism, cells continuously consume energy and nutrients, while producing new energy and nutrients [31]. Similarly, our skeletal system has a continuous remodeling cycle, and an appropriate balance between anabolism and catabolism is needed to maintain the strength and healthy microstructure of bone tissue [32]. Bone remodeling is accomplished through the coordinated efforts of four key cells: bone marrow mesenchymal stem cells (MSCs) which are the source of osteoblasts (OBs) and exert regulatory functions throughout remodeling; OBs are located on the bone surface and secrete bone matrix; matrix-embedded OBs further differentiate into persistent osteocytes (OCT), which form a mechanosensory network in bone and play a crucial role in paracrine signaling. At the same time, osteoclasts (OC) continuously degrade and absorb the surrounding bone base [33]. The dynamic balance between bone formation and bone resorption is continuously coordinated. As ferroptosis is an important mode of regulated cell death, its relationship with skeletal cells, including MSCs, OBs, OCs, and OCTs, has attracted attention in recent decades [34].

### 3.1. Ferroptosis and the MSCs

Recent research in the study of bone tissue repair and regeneration has paid particular attention to MSCs. MSCs have the potential of multidirectional differentiation with low immunogenicity and wide availability. They can migrate to damaged tissues and organs to reconstruct these through direct differentiation or secretion of exosomes, growth factors, and cytokines [35, 36]. Furthermore, the regulatory role of MSCs in ameliorating cell ferroptosis has been well-documented [37]. For example, it was recently shown that MSCs are capable of inhibiting the production of lipid peroxidation and alleviating ferroptosis both *in vitro* and *in vivo*. The authors also demonstrated that MSC-derived exosomes are involved in the underlying mechanisms of the effect of MSCs on ferroptosis, which could significantly downregulate the expression of prostaglandin-endoperoxide synthase 2 and promote SLC7A11 expression [38]. Similarly, the suppressive effect of MSCs on ferroptosis was seen in neuronal cells [39]. In an acute spinal cord injury mouse model, researchers demonstrated that MSCs and their exosomes could ameliorate spinal cord injury through promotion of the expression of ferroptosis inhibitor (FSP1) [39]. In addition to the discovery of the antiferroptotic effect of MSCs, the underlying mechanism of ferroptosis in MSCs was also investigated. It is well-documented that NOP2/Sun RNA methyltransferase 5 (NSUN5) posttranscriptionally can mediate ferroptosis in MSCs through RNA methylation [40]. A recent study further found that NSUN5 is downregulated in erastin-induced ferroptosis in MSCs, while NSUN5 is capable of suppressing ferritin heavy chain/light-chain (FTH1/FTL) activity. In the NSUN5 depletion experiments, they found an accumulation of intracellular iron and a marked decrease of GPX4, suggesting that the NSUN5-FTH1/FTL pathway mediates ferroptosis in MSCs and that therapeutic targeting of components of this pathway may promote resistance to ferroptosis and improve the survival of MSCs [40].

### 3.2. Ferroptosis and OBs

The integrity of bone is maintained through an appropriate balance between osteogenic and osteoclastic activities, and the bone remodeling process is a continuous cycle. OBs are mainly involved in bone reconstruction, including formation, mineralization, and construction of osteocytes [41]. A variety of studies have focused on the potential mechanisms and agents regulating OB ferroptosis [42]. Advanced glycation end products were recently found to induce OB ferroptosis and promote osteoporosis [43]. Inversely, melatonin, a hormone secreted by the pineal gland, was shown to ameliorate OB ferroptosis and enhance the osteogenic capacity of OB via activation of Nrf-2/HO-1 signaling [44]. Mechanistically, mitochondrial ferritin (FtMt) was reported to exert a critical role in regulating cell ferroptosis via storing iron ions and intercepting toxic ferrous ions in mitochondria [45]. The researchers found that activation of FtMt could ameliorate OB ferroptosis while inhibition of FtMt could induce mitophagy through ROS/PINK1/Parkin signaling [45]. Moreover, increased ferroptosis in OBs could be seen after activating mitophagy, with the findings suggesting that FtMt can effectively suppress ferroptosis in OBs [45]. Interestingly, exosomes, extracellular vesicles containing active regulatory factors, have been shown to participate in the regulation of ferroptosis in OB. For example, one recent study reported that vascular endothelial cells could effectively prevent osteoblastic ferroptosis through exosome release which could further suppress ferritinophagy and limit ferroptosis of OBs [46]. Similarly, using an osteoporotic murine model, it was reported that exosomes from endothelial progenitor cells could inhibit steroid-induced osteoporosis through suppression of the ferroptotic pathway [47].

### 3.3. Ferroptosis and OCs

Iron ions is capable to induce OC differentiation and bone resorption through the production of ROS [48, 49]. Zoledronic acid (ZA), a bisphosphonate, has been reported to inhibit OC growth via induction of ferroptosis of the OC [49]. The role of ZA in regulating osteoclast function was evaluated using a RANKL-induced cell model, which indicated that ZA treatment suppressed the cell viability of osteoclasts and facilitated osteoclast ferroptosis with an increase in iron ions and ROS and decrease in the GPX4 and GSH level [49]. Similarly, ferroptosis was reported involved in OC function during RANKL-induced differentiation and is induced by iron starvation response and ferritin phagocytosis [50]. Mechanically, subsequent RANKL stimulation can lead to iron droop due to iron starvation response (increased transferrin receptor 1 and decreased ferritin) under normoxic but not hypoxic conditions, due to downregulation of aconitase activity [50]. Based on these results, it can be assumed that ferroptosis of OCs can limit bone resorption, while inducing ferroptosis in OCs could be an alternative treatment of disorders of bone formation.

### 3.4. Ferroptosis and OCTs

OCTs, the most prevalent cells in mineralized bone tissue, communicate with other bone cells, such as OBs and OCs, via the lacunar-canalicular system and through various secreted hormones [51]. Decreased activity and death of OCTs induced by internal and external factors can lead to bone loss and destruction of bone microstructure. Therefore, effective promotion of OCT survival is a promising therapeutic strategy for maintenance of bone homeostasis. It has been reported that ferroptosis is an important form of OCT death, which can be reversed by targeting the inhibition of ferroptosis signaling pathways [52]. Yang et al. [53] found that a hyperglycemic microenvironment is capable of promoting lipid peroxidation and iron overload, thereby inducing osteocyte ferroptosis. Furthermore, RNA sequencing results indicated that heme oxygenase-1 (HO-1) is overexpressed in ferroptotic osteocytes, suggesting that HO-1 is essential for osteocyte ferroptosis. Similarly, a recent study reported that dexamethasone could notably induce ferroptosis in MC3T3-E1 cells (a type of OCT precursor cell) via downregulation of the p53/SLC7A11/GPX4 signaling pathway, providing a potential mechanism for the effect of ferroptosis on osteocytes in steroid- (glucocorticoid) induced osteonecrosis of the femoral head [54]. These findings highlight a potential therapeutic target for the treatment of skeletal disorders.

## 4. Ferroptosis and Bone Degenerative Disorders

Ferroptosis differs from apoptosis, autophagy, necrosis, and pyrodeath, in that it mainly involves iron metabolism and lipid peroxidation. Ferroptosis plays an important role in malignant tumors, cardiovascular diseases, and neural system diseases [55, 56]. Iron overload is closely related to cellular ferroptosis, and iron overload and lipid peroxide accumulation jointly mediate bone destruction, ultimately leading to bone disorders.

### 4.1. Ferroptosis and Osteoporosis

Osteoporosis is a systemic bone disease characterized by a reduction in bone mass and a degeneration of the fibrous structure of bone tissue, which results in increased bone brittleness and risk of fracture [57]. Its pathological features include the following [58]: (1) decreased bone mass, including a reduction in the proportion of bone minerals and other substrates; (2) degeneration of bone microstructure, caused by absorption and imbalance of bone tissue homeostasis, manifesting as destruction, deformation, and fracture of bone trabecular structure; and (3) increased bone brittleness and decreased bone strength, increased fracture deformation, decreased load bearing force, and more frequent microfracture or complete fracturing. Iron is a strong oxidant that can promote the production of ROS radicals, and iron metabolism can directly or indirectly affect the occurrence and development of type 2 diabetes [59, 60]. Ferroptosis results in the production of abundant ROS through the Fenton reaction, inducing accumulation of lipid peroxides and cell damage [61]. It was well-documented that hyperglycemia can induce ferroptosis in the bone tissue of an osteoporotic rat model by production of ROS/lipid peroxidation. Melatonin was shown to ameliorate the level of ferroptosis through activation of Nrf2/HO-1 signaling and promotion of the osteogenic differentiation of MC3T3-E1 cells [44]. Similarly, in a murine model of diabetic osteoporosis (DOP), the researchers verified the important role of ferroptosis in DOP-induced OCT death. Mechanistically, activation of Nrf2/HO-1 signaling could lead to lipid peroxidation and cell ferroptosis, suggesting that targeting inhibition of OCT ferroptosis may be a potential therapeutic strategy for DOP treatment [53].

Furthermore, the relationship between ferroptosis and glucocorticoid-induced osteoporosis (GIOP) has been well investigated [62]. For example, a recent study [47] reported that high-dose dexamethasone (10 *μ*M) can induce ferroptosis of OB by inhibiting the expression of GPX4 and system Xc^−^. To investigate the underlying mechanisms, extracellular vesicles were extracted from bone marrow-derived endothelial progenitor cells (EPC-EVs), which were seen to suppress ferroptosis by restoring the activity of GPX4 and system Xc^−^. Significantly, EPC-EVs were capable of reversing dexamethasone-induced changes in cysteine and oxidative damage markers and improved skeletal parameters in mice. These results suggest that EPC-EVs reverse murine GIOP through inhibition of OB ferroptosis.

### 4.2. Ferroptosis and Osteoarthritis (OA)

**Figure 2:**
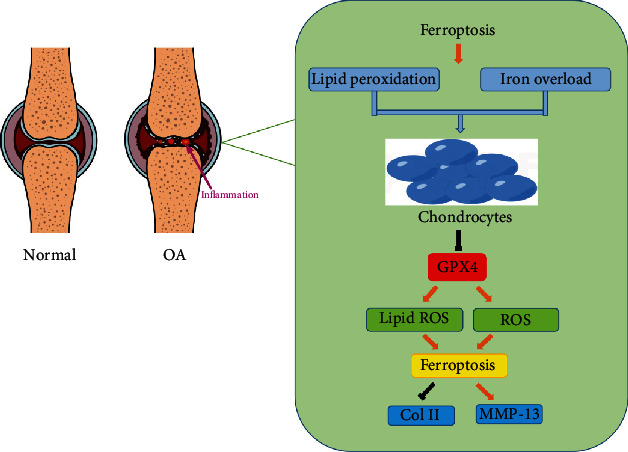
The potential mechanism of ferroptotic chondrocytes.

OA is a degenerative disease characterized by the pathological alteration of the function and morphology of an entire joint, as well as articular cartilage destruction and damage to other joint components [63–66]. Generally, OA occurs due to chronic heavy loading and biomechanical damage; however, pathological progress at the molecular level has also been proposed in the development of OA. Therefore, maintaining chondrocytes in a healthy state is considered to be an effective strategy for preserving the integrity of the entire cartilage [67–70]. It can be, therefore, assumed that ferroptosis may be involved in the progression of OA. In a recent study [71], researchers used interleukin-1 beta (IL-1*β*) to construct an *in vitro* iron-overload model. The authors found that IL-1*β* could induce both ROS and lipid ROS accumulations and saw ferroptosis-related protein expression changes in the chondrocytes. Furthermore, increased MMP13 expression and decreased collagen II expression were seen in the ferroptotic chondrocytes (Figure 2). In a murine OA model, intra-articular injection of a ferroptosis inhibitor was seen to prevent OA progression. These findings highlight the contribution of ferroptosis in chondrocytes to the progression of OA. Studies have also been conducted to identify a feasible treatment for chondrocyte degeneration with a focus on cell ferroptosis [72]. Deferoxamine (DFO) [73] and D-mannose were recently demonstrated to alleviate OA progression by inhibiting of chondrocyte ferroptosis. DFO was found to both effectively ameliorate chondrocyte ferroptosis and induce activation of the Nrf2 antioxidant system, which is crucial for chondrocyte protection [73]. The efficacy of injection of DFO in OA mice was also demonstrated *in vivo* [73]. Similarly, Zhou et al. [74] investigated whether D-mannose mediates chondrocyte ferroptosis during OA cartilage degeneration *in vitro* and *in vivo*. They found that D-mannose could exert a chondroprotective effect by attenuating the sensitivity of chondrocytes to ferroptosis and could alleviate OA progression. Furthermore, HIF-2*α* was identified as a central mediator in the D-mannose-induced resistance of chondrocytes to ferroptosis. These findings provide potential therapeutic strategies for ferroptosis-related bone diseases.

## 5. Conclusion

During the past decades, there has been an accumulative research focus on the relationship between ferroptosis and diseases [75, 76]. The significance of ferroptosis in cell survival and differentiation is widely accepted, and its regulatory role in the modulation and treatment of diverse disease has been gradually uncovered [77, 78]. However, there are still some academic problems yet to be resolved. For example, the relationship between ferroptosis and the other forms of regulated cell death in the regulation of skeletal disorders should be further revealed. Furthermore, the detailed molecular mechanisms that activate ferroptosis are still unascertained. In addition, plenty of emerging evidences have demonstrated that exosomes are involved in the modulation of ferroptosis and skeletal disease [79, 80]. In-depth knowledge of this exosome-mediated effect should be achieved by performing more researches regarding the crosstalk between exosome and ferroptosis.

The recent continuous efforts in the research of ferroptosis have shed light on the interaction between ferroptosis and bone homeostasis. Technical limitations currently restrict the in-depth understanding of the mechanisms underlying ferroptotic regulation. Specifically, lack of an effective and specific ferroptotic blocker precludes the observation of the effect of blockade on the physiological functions of ferroptosis in *in vivo* models. Furthermore, lack of defined and specific ferroptotic signaling pathways or biomarkers hinders the verification of ferroptosis in physiological or pathological conditions. In addition, given the limitations of existing experimental techniques, we do not yet have a visual reporting method of *in vivo* ferroptosis detection. In future studies of ferroptosis, research should focus on in-depth study of the molecular mechanisms underlying ferroptosis and screening and identifying specific signaling pathways. Furthermore, efforts should be invested in developing a feasible detectable tool for measuring ferroptosis *in vivo*. Finally, the regulatory role of ferroptosis in the process of bone aging should be elucidated.

## Figures and Tables

**Table 1:** The comparative characteristics among ferroptosis, apoptosis, and autophagy.

RCD	Ferroptosis	Apoptosis	Autophagy
Hallmarks	Mitochondrial crest disappeared; mitochondrial outer membrane rupture and shrinkage; mitochondria are deeply stained	Condensation and fragmentation of chromatin; nucleoli disappeared; nuclear pyknosis and fragmentation	Autophagy lysosome formation
Other characteristics	No nuclear rupture; cell membrane rupture	Cell shrinkage; the outflow of the cytoplasm and vacuolation of membrane	No changes in nuclear and cell membrane
Biomarkers	Upregulated: ROS, PTGS2; downregulated: NADPH	Cytochrome C releases caspase-activated intracellular calcium increases	Transformation from LC3-I into LC3-II
Positive regulators	Erastin, RSL3, RAS, Sorafenib, p53	P53, Bax, Bak, TGF-B, radiation	ATG family, Beclin1
Negative regulators	GPX4, FSP1, SLC7A11, NRF2, Ferrostatin-1, Liproxstatin-1, DFO	Bcl-2, Bcd-XL, Z-VAD-FMK	3-Methyladenine, Wortmannin, Spautin1

RCD: regulated cell death; PTGS2: prostaglandin endoperoxide synthase 2; FSP1: fibroblast-specific protein 1; NRF2: nuclear factor erythroid 2-related factor 2; DFO: desferrioxamine.

## Data Availability

The data used to support the findings of this study are available from the corresponding author upon request.
